# Balancing Accuracy and Readability: Comparative Evaluation of AI Chatbots for Patient Education on Rotator Cuff Tears

**DOI:** 10.3390/healthcare13212670

**Published:** 2025-10-23

**Authors:** Ali Can Koluman, Mehmet Utku Çiftçi, Ebru Aloğlu Çiftçi, Başar Burak Çakmur, Nezih Ziroğlu

**Affiliations:** 1 Department of Orthopedics and Traumatology, Bakirkoy Dr. Sadi Konuk Training and Research Hospital, 34147 Istanbul, Turkey; alican.koluman@saglik.gov.tr; 2 Department of Orthopedics and Traumatology, Sultan Abdülhamid Han Training and Research Hospital, 34668 Istanbul, Turkey; mehmetutkuciftci.md@gmail.com; 3 Faculty of Health Sciences, Division of Physiotherapy and Rehabilitation, Istanbul Okan University, 34959 Istanbul, Turkey; ebru.aloglu@okan.edu.tr; 4 Department of Physiotherapy and Rehabilitaation, Institute of Graduate Education, Istinye University, 34010 Istanbul, Turkey; 5 Department of Orthopedics and Traumatology, Beylikduzu State Hospital, 34500 Istanbul, Turkey; basar_cakmur@hotmail.com; 6 Department of Orthopedic Prosthetics and Orthotics, Vocational School of Health Services, Acıbadem Mehmet Ali Aydınlar Üniversitesi, 34638 Istanbul, Turkey; 7 Department of Orthopaedics and Traumatology, Acıbadem Üniversitesi Atakent Hospital, 34303 Istanbul, Turkey

**Keywords:** rotator cuff injuries, artificial intelligence, chatbots, large language models, patient education, health literacy, digital health

## Abstract

Background/Objectives: Rotator cuff (RC) tears are a leading cause of shoulder pain and disability. Artificial intelligence (AI)-based chatbots are increasingly applied in healthcare for diagnostic support and patient education, but the reliability, quality, and readability of their outputs remain uncertain. International guidelines (AMA, NIH, European health communication frameworks) recommend that patient materials be written at a 6th–8th grade reading level, yet most online and AI-generated content exceeds this threshold. Methods: We compared responses from three AI chatbots—ChatGPT-4o (OpenAI), Gemini 1.5 Flash (Google), and DeepSeek-V3 (Deepseek AI)—to 20 frequently asked patient questions about RC tears. Four orthopedic surgeons independently rated reliability and usefulness (7-point Likert) and overall quality (5-point Global Quality Scale). Readability was assessed using six validated indices. Statistical analysis included Kruskal–Wallis and ANOVA with Bonferroni correction; inter-rater agreement was measured using intraclass correlation coefficients (ICCs). Results: Inter-rater reliability was good to excellent (ICC 0.726–0.900). Gemini 1.5 Flash achieved the highest reliability and quality, ChatGPT-4o performed comparably but slightly lower in diagnostic content, and DeepSeek-V3 consistently scored lowest in reliability and quality but produced the most readable text (FKGL ≈ 6.5, within the 6th–8th grade target). None of the models reached a Flesch Reading Ease (FRE) score above 60, indicating that even the most readable outputs remained more complex than plain-language standards. Conclusions: Gemini 1.5 Flash and ChatGPT-4o generated more accurate and higher-quality responses, whereas DeepSeek-V3 provided more accessible content. No single model fully balanced accuracy and readability. Clinical Implications: Hybrid use of AI platforms—leveraging high-accuracy models alongside more readable outputs, with clinician oversight—may optimize patient education by ensuring both accuracy and accessibility. Future work should assess real-world comprehension and address the legal, ethical, and generalizability challenges of AI-driven patient education.

## 1. Introduction

Rotator cuff (RC) tears are the most common tendon injuries of the shoulder and represent a major cause of pain and disability, particularly in middle-aged and older adults [1,2]. The incidence of RC tears increases with age, contributing to substantial functional impairment and reduced quality of life [3]. Advances in surgical and non-surgical management have improved patient outcomes; however, treatment success also depends heavily on effective patient education, informed decision-making, and realistic postoperative expectations.

Patients frequently seek health-related information online before or after clinical consultations. Although digital platforms can provide rapid access to medical knowledge, previous evaluations of online materials regarding RC repair have demonstrated variable quality, outdated content, and language complexity beyond the comprehension level of the general population [4,5,6]. High-quality patient education resources authored by healthcare professionals or academic institutions have been shown to be more accurate and reliable, but such resources remain less accessible compared to general internet content [7]. Importantly, international guidelines such as those from the American Medical Association (AMA) and the National Institutes of Health (NIH) recommend that patient education materials be written at a 6th to 8th grade reading level, while European health communication frameworks emphasize “plain language” to ensure accessibility across diverse populations [8,9,10]. Despite these recommendations, most online and AI-generated medical content continues to exceed the recommended readability thresholds [5,6,9].

Artificial intelligence (AI)-based conversational models, such as large language models (LLMs), are increasingly being explored for medical applications, including diagnosis, decision support, and patient education [10,11]. Recent studies in cardiology, palliative care, and endocrinology have revealed that LLMs can generate clinically relevant information, but often at readability levels above the recommended standard [12,13,14]. Conversely, Will et al. demonstrated that advanced AI models are capable of simplifying medical texts to patient-appropriate levels without compromising accuracy, underscoring their potential as tools for health literacy improvement [15]. In addition, some studies reviewed how AI technologies can improve disease diagnosis by enhancing accuracy and reducing diagnostic delays, particularly through imaging-based applications [11,16].

In orthopedics, recent work has evaluated AI-generated responses on conditions such as scoliosis and rotator cuff repair, highlighting variability in reliability, readability, and quality across different chatbot platforms [10,17]. Other studies have also demonstrated that AI can accurately identify RC tears from imaging [18] and generate patient-directed educational content [14,17,19]. However, critical aspects such as factual accuracy, completeness, and readability remain insufficiently studied, and few investigations have directly compared multiple models in this specific context.

Given that RC tears present complex diagnostic and therapeutic considerations, it is essential to ensure that AI-generated information meets the needs of patients with diverse educational backgrounds. The ability to deliver accurate, high-quality, and understandable information could enhance patient–physician communication, support shared decision-making, and potentially improve treatment adherence.

Therefore, this study aimed to systematically compare the reliability, usefulness, quality, and readability of three widely used AI chatbot models—ChatGPT-4o (OpenAI), Gemini 1.5 Flash (Google DeepMind), and DeepSeek-V3 (Deepseek AI)—in providing responses to frequently asked patient questions regarding RC tears. To our knowledge, this is the first head-to-head comparison of multiple AI chatbot models in orthopedics that jointly evaluates reliability, quality, and readability in patient-directed educational content. By evaluating AI outputs against orthopedic expert consensus, this work seeks to identify the strengths and limitations of each model and inform the development of optimized, AI-driven patient education tools.

## 2. Materials and Methods

### 2.1. Study Design

This cross-sectional comparative study evaluated the reliability, usefulness, quality, and readability of responses generated by three large language model (LLM)-based AI chatbots—ChatGPT-4o (OpenAI), Gemini 1.5 Flash (Google), and DeepSeek-V3 (Deepseek AI)—to patient-directed questions about rotator cuff (RC) tears. The study design followed a multi-phase process comprising question selection, AI response collection, expert evaluation, and readability analysis.

### 2.2. Question Development

Initially, each AI chatbot was prompted with the query: “What are the most frequently asked questions by patients about shoulder tendon tears?” Across the three chatbots, an initial pool of 44 questions was generated. After removing duplicates and consolidating semantically overlapping items (e.g., “What is a rotator cuff tear?” vs. “What does it mean to have a torn rotator cuff?”), we excluded questions that were either contextually irrelevant to RC pathology or infrequently represented in clinical practice and Google Trends analysis. This refinement yielded a final set of 20 questions.

The number of questions was selected to balance comprehensiveness with feasibility, ensuring adequate coverage of patient concerns across diagnostic, treatment, and lifestyle domains while maintaining a manageable workload for expert raters. Similar ranges (15–25 questions) have been adopted in prior AI evaluation studies in orthopedics and other medical fields [10,11,14], supporting the adequacy of this sample size.

The final 20 questions (Table 1) were categorized into three thematic domains: diagnosis and symptoms (*n* = 7), treatment and interventions (*n* = 10), and lifestyle and activity (*n* = 3). Four board-certified orthopedic surgeons independently reviewed the list for content validity and approved the final wording.

### 2.3. AI Response Collection

Each of the 20 questions was entered separately into the respective AI chatbots by an independent researcher not involved in the scoring process. Responses were saved in their original form in Microsoft Word (version 16.98, Microsoft Corp., Redmond, WA, USA). To minimize evaluator bias, all responses were anonymized and coded before assessment.

### 2.4. Expert Evaluation

Four board-certified orthopedic surgeons independently assessed each AI-generated response. Before evaluation, the raters participated in a calibration session in which the definitions and scoring criteria for Reliability, Usefulness, and Global Quality Scale (GQS) were reviewed using sample responses not included in the main dataset. Written instructions were also provided to standardize scoring and ensure reproducibility across raters. Evaluation criteria included: Reliability (7-point Likert; 1 = completely unreliable, 7 = absolutely reliable), Usefulness (7-point Likert; 1 = not useful at all, 7 = extremely useful), and Overall Quality (5-point GQS, where higher scores indicate more comprehensive and well-structured information).

### 2.5. Readability Analysis

Readability was evaluated using six established indices: Flesch Reading Ease (FRE), Flesch-Kincaid Grade Level (FKGL), Gunning Fog Index (GFI), Coleman–Liau Index (CLI), Simple Measure of Gobbledygook (SMOG), and Automated Readability Index (ARI). These indices were chosen because they are widely used in health communication research, collectively capture different dimensions of textual complexity (sentence length, word length, syllable count, and vocabulary difficulty), and allow comparisons against recommended readability standards for patient education (e.g., 6th–8th grade level in the U.S. and plain language principles in Europe). Calculations were performed using the online platform Readable.com (United Kingdom). Higher FRE scores indicate easier readability, whereas higher FKGL, GFI, CLI, SMOG, and ARI scores denote greater reading complexity.

### 2.6. Statistical Analysis

All statistical analyses were performed using IBM SPSS Statistics 27.0. Descriptive statistics were reported as mean ± standard deviation (SD), median, and interquartile range (IQR). Normality of continuous variables was assessed with the Shapiro–Wilk test. Inter-rater reliability was estimated using the intraclass correlation coefficient (ICC) with a two-way random-effects, absolute-agreement model. Non-normally distributed Likert-scale data were analyzed using the Kruskal–Wallis test, with Bonferroni–Dunn-adjusted Mann–Whitney U tests for pairwise comparisons. Normally distributed readability metrics were analyzed with one-way ANOVA with Bonferroni-adjusted post hoc tests where applicable. Effect sizes were reported as eta squared (η^2^); statistical significance was set at *p* < 0.05.

### 2.7. Model Versioning and Access Dates

We accessed ChatGPT-4o (OpenAI), Gemini-2.5-Flash (Google), and DeepSeek-V3 (DeepSeek AI) between July and August 2025. All prompts were entered via the official web user interfaces; browsing or plug-ins were disabled. No regenerative sampling beyond the first complete response was allowed unless a model produced an explicit refusal.

### 2.8. Rating Scales

Reliability and usefulness were scored on 7-point Likert-type scales as originally described by Likert and commonly applied in health research, while overall quality was assessed with the 5-point Global Quality Scale (GQS).

### 2.9. Inter-Rater Agreement Reporting

Intraclass correlation coefficients (ICC) and 95% confidence intervals were calculated using a two-way random-effects, absolute-agreement model following contemporary reporting recommendations.

## 3. Results

### 3.1. Inter-Rater Reliability

Inter-rater agreement among the four orthopedic surgeons was good to excellent across all evaluation criteria, with ICC values ranging from 0.726 to 0.900 (Table 2). For reliability, ChatGPT-4o demonstrated the highest ICC (0.900), followed closely by Gemini-2.5-Flash (0.897), whereas DeepSeek-V3 had the lowest (0.755). In terms of usefulness, both ChatGPT-4o (ICC = 0.848) and DeepSeek-V3 (ICC = 0.822) showed excellent agreement, while Gemini 1.5 Flash achieved good agreement (ICC = 0.776). For the Global Quality Scale (GQS), Gemini 1.5 Flash achieved the highest inter-rater agreement (ICC = 0.856). According to established thresholds, ICC values ≥ 0.75 indicate good agreement and ≥0.90 indicate excellent agreement.

### 3.2. Reliability Scores

When comparing reliability scores across the three clinical domains (diagnosis/symptoms, treatment/intervention, and lifestyle/activity), statistically significant differences were observed for diagnosis/symptoms (*p* = 0.002) and treatment/intervention (*p* = 0.001) (Table 3).

- Diagnosis/Symptoms: Gemini 1.5 Flash scored significantly higher than both ChatGPT-4o (*p* = 0.045) and DeepSeek-V3 (*p* = 0.003).
- Treatment/Intervention: DeepSeek-V3 scored significantly lower than both Gemini 1.5 Flash and ChatGPT-4o (*p* < 0.005), with no difference between the latter two (*p* = 0.072).
- Lifestyle/Activity: No significant differences were observed (*p* = 0.057).

**Figure 1:**
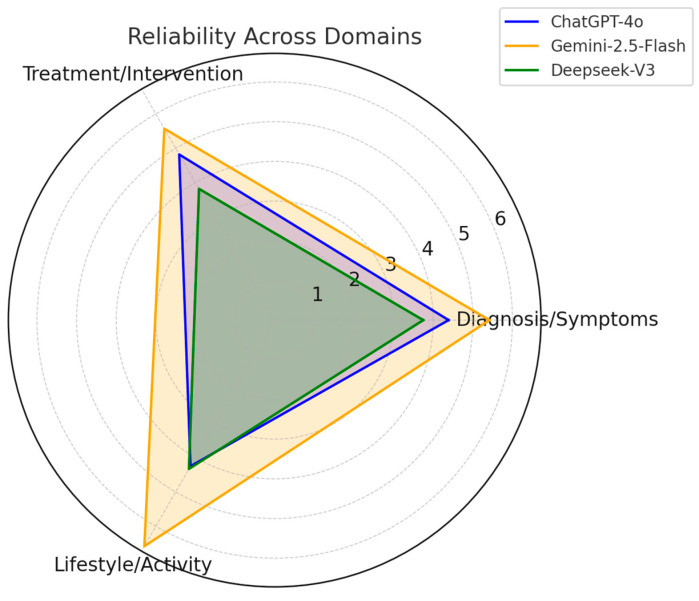
Reliability scores of ChatGPT-4o, Gemini 1.5 Flash, and DeepSeek-V3 across the three clinical domains (diagnosis/symptoms, treatment/intervention, lifestyle/activity). Gemini demonstrated consistently higher reliability in diagnosis and treatment, whereas differences were smaller in lifestyle/activity.

Overall, Gemini 1.5 Flash achieved the highest mean reliability (5.67 ± 0.72), followed by ChatGPT-4o (4.58 ± 0.67) and DeepSeek-V3 (3.87 ± 0.46) (*p* = 0.001) (Figure 1).

### 3.3. Usefulness Scores

Usefulness ratings also differed significantly in the diagnosis/symptoms and treatment/intervention domains, as well as overall scores (*p* < 0.05) (Table 3).

- Gemini 1.5 Flash and ChatGPT-4o both received higher scores compared with DeepSeek-V3 in diagnosis and treatment domains.
- In the lifestyle/activity domain, no significant differences were observed (*p*-adj > 0.05).

**Figure 2:**
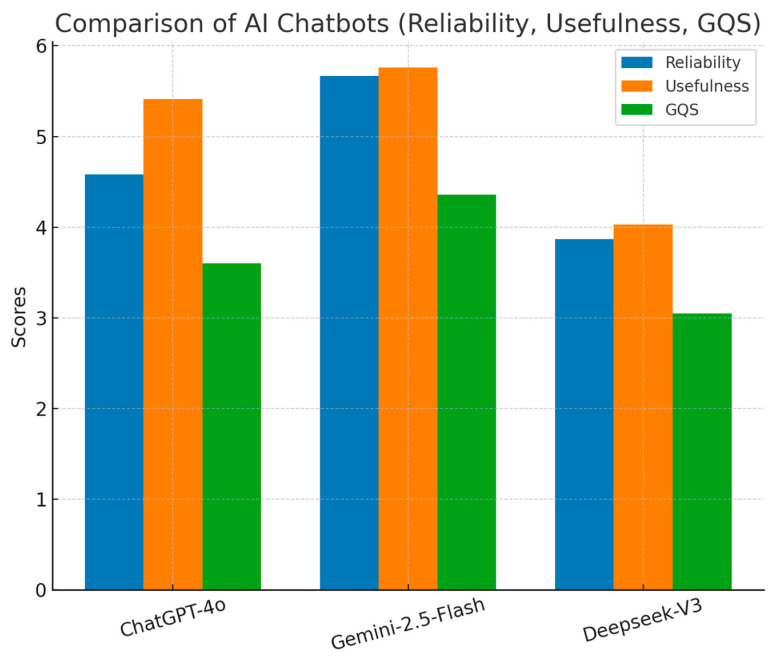
Comparison of overall reliability, usefulness, and Global Quality Scale (GQS) scores for ChatGPT-4o, Gemini 1.5 Flash, and DeepSeek-V3. Higher scores indicate better performance.

Overall usefulness scores were highest for Gemini 1.5 Flash (5.76 ± 0.44), followed by ChatGPT-4o (5.41 ± 0.50) and DeepSeek-V3 (4.03 ± 0.51) (*p* = 0.001) (Figure 2).

### 3.4. Global Quality Scale (GQS) Scores

GQS scores also showed significant differences across models in diagnosis/symptoms (*p* = 0.001) and treatment/intervention (*p* = 0.005) domains (Table 3).

- Gemini 1.5 Flash achieved the highest GQS in diagnosis/symptoms (4.53 ± 0.26), significantly outperforming both ChatGPT-4o (*p* = 0.003) and DeepSeek-V3 (*p* = 0.003).
- In treatment/intervention, Gemini 1.5 Flash scored significantly higher than DeepSeek-V3 (*p* = 0.018), while no significant difference was observed between ChatGPT-4o and Gemini 1.5 Flash.
- No differences were detected in lifestyle/activity (*p* = 0.050).

Overall, Gemini 1.5 Flash achieved the highest GQS (4.36 ± 0.58), followed by ChatGPT-4o (3.60 ± 0.36) and DeepSeek-V3 (3.05 ± 0.37) (*p* = 0.001).

Flesch Reading: Higher scores indicate better reliability, usefulness, or quality. GQS = Global Quality Scale. *p*-values are Bonferroni-adjusted; only significant pairwise differences are reported in the table. Gemini 1.5 Flash generally outperformed the other models in reliability and quality, whereas ChatGPT-4o performed at an intermediate level and DeepSeek-V3 consistently scored lowest.

### 3.5. Readability

Significant differences were observed across all six readability indices (*p* < 0.001) (Table 4).

- Flesch Reading Ease (FRE): DeepSeek-V3 had the highest score (45.9 ± 7.6), indicating the most patient-friendly readability, followed by ChatGPT-4o (37.0 ± 7.3) and Gemini 1.5 Flash (30.7 ± 7.3).
- Grade-Level and Complexity Indices (FKGL, GFI, CLI, SMOG, ARI): Gemini 1.5 Flash consistently produced the most complex text (FKGL ≈ 11.6), DeepSeek-V3 generated the simplest responses (FKGL ≈ 6.5), while ChatGPT-4o scored in between (FKGL ≈ 8.9).

These findings highlight an inverse relationship between accuracy/quality and readability: Gemini 1.5 Flash generated the most reliable and highest-quality responses, but at the expense of higher reading difficulty, whereas DeepSeek-V3 produced simpler text closer to the recommended 6th–8th grade level but with lower reliability and quality. Specifically, DeepSeek-V3 achieved a Flesch-Kincaid Grade Level (FKGL) of 6.5, which falls within the recommended 6th–8th grade range for patient education, while ChatGPT-4o (FKGL 8.9) and Gemini 1.5 Flash (FKGL 11.6) both exceeded this threshold. Moreover, none of the models reached a Flesch Reading Ease (FRE) score above 60, indicating that even the most readable outputs remain more complex than the ideal plain-language standard.

Higher FRE scores indicate easier readability (recommended ≥60 for lay readers). Higher FKGL, GFI, CLI, SMOG, and ARI scores indicate greater reading difficulty (recommended ≤6–8th grade level for patient education). DeepSeek-V3 generated text closest to the recommended readability level, Gemini consistently produced the most complex text, and ChatGPT-4o showed intermediate performance. *p*-values are Bonferroni-adjusted; only significant pairwise comparisons are shown.

## 4. Discussion

This study provides a direct, multi-criteria comparison of three widely used AI chatbot models—ChatGPT-4o, Gemini 1.5 Flash, and DeepSeek-V3—in generating patient-oriented information on rotator cuff (RC) tears. To our knowledge, this is the first head-to-head evaluation in orthopedics that jointly assesses reliability, usefulness, overall quality, and readability. Our findings reveal an inherent trade-off: while Gemini 1.5 Flash and ChatGPT-4o generally produced more accurate and higher-quality content, DeepSeek-V3 generated responses with superior readability, closer to recommended health literacy standards.

### 4.1. Reliability and Usefulness of AI-Generated Information

Gemini 1.5 Flash achieved the highest overall reliability, particularly in diagnosis/symptoms and treatment/intervention domains, with statistically significant superiority over ChatGPT-4o and DeepSeek-V3. This aligns with prior work demonstrating that model architecture, training data recency, and domain coverage influence factual accuracy in medical topics [8,13]. ChatGPT-4o performed comparably to Gemini 1.5 Flash for treatment-related questions but trailed slightly in diagnostic explanations, suggesting subtle differences in clinical reasoning capabilities. DeepSeek-V3 consistently scored lower in both reliability and usefulness, reflecting potential limitations in its medical knowledge base or response generation algorithms.

Usefulness ratings paralleled these findings, with Gemini 1.5 Flash and ChatGPT-4o outperforming DeepSeek-V3. These results are consistent with prior studies in other specialties showing that LLMs can provide patient-friendly, clinically applicable information provided that outputs are accurate [10,14]. However, even the best-performing models occasionally produced incomplete or generalized statements, highlighting the need for clinician oversight in patient counseling.

### 4.2. Quality Assessment

Global Quality Scale (GQS) scores reflected the comprehensiveness, organization, and clarity of chatbot outputs. Gemini 1.5 Flash consistently achieved the highest GQS ratings, particularly in diagnostic and treatment domains, followed by ChatGPT-4o. DeepSeek-V3 ranked lowest, echoing recent evaluations of AI-generated orthopedic content that reported wide variability in structural quality [10,13]. The relatively higher GQS scores of Gemini 1.5 Flash may be attributed to its more logically sequenced and structured responses.

### 4.3. Readability Considerations

A novel and clinically important finding was the inverse relationship between accuracy/quality and readability. DeepSeek-V3 generated the most readable responses across all six indices, particularly the Flesch Reading Ease (FRE), producing text closer to the 6th–8th grade reading level recommended by the AMA and NIH. By contrast, Gemini 1.5 Flash produced more complex outputs despite superior accuracy and quality. Similar discrepancies between quality and readability have been documented in online orthopedic materials, where higher-quality sources often exceeded recommended literacy thresholds [5,6]. Importantly, while DeepSeek-V3 was the only model to produce text within the recommended FKGL target range, its FRE score (45.9) remained below the plain-language threshold of 60. This suggests that even the most readable AI-generated content may still present challenges for patients with limited health literacy. Our results reinforce this challenge and suggest that layered information delivery—providing a plain-language summary alongside a more detailed version—may represent a practical strategy for bridging the gap.

### 4.4. Clinical and Digital Health Implications

From a clinical perspective, the combination of accuracy and readability is essential for effective patient education. No single model currently provides both optimally, implying that a hybrid approach—leveraging Gemini 1.5 Flash for accuracy and DeepSeek-V3 for readability—may serve as a pragmatic interim solution. These outputs, however, must be reviewed and adapted by clinicians to ensure appropriateness for patients with varying literacy levels.

Beyond clinical integration, legal and ethical considerations are critical. Liability for misinformation, patient misinterpretation without professional guidance, and data privacy represent significant risks. Regulatory frameworks remain underdeveloped, raising questions about accountability when AI-generated advice influences patient decisions. Until such safeguards are established, clinician oversight and institutional governance are indispensable. In addition to these issues, AI systems in healthcare face significant security and privacy challenges. Potential risks include patient data leakage, unauthorized access, adversarial attacks, and difficulties in ensuring compliance with regulatory frameworks such as GDPR in Europe and HIPAA in the United States. Addressing these vulnerabilities—through robust governance, security audits, and privacy-preserving or federated learning approaches—is essential to safeguard patient trust and enable responsible deployment of AI tools in clinical care [20,21,22].

### 4.5. Limitations and Future Directions

This study has several limitations. First, it evaluated only three chatbot models, and performance may differ across other platforms or future versions of the same models. Second, the analysis was limited to RC tear-related questions; therefore, findings may not be generalizable to other musculoskeletal or non-orthopedic conditions. Third, the evaluation was conducted by a relatively small group of four orthopedic surgeons, which may limit external validity despite the good inter-rater reliability observed. Fourth, readability metrics measure textual complexity but not actual patient comprehension. We did not perform comprehension testing with real patients, which represents a critical limitation; future studies should validate chatbot outputs against real-world understanding and decision-making outcomes. Finally, AI models evolve rapidly, and updates may substantially alter performance within weeks, making our results a snapshot rather than a permanent benchmark. This rapid progress is largely driven by advances in automated machine learning (AutoML), which can optimize model selection and hyperparameters with minimal human intervention. Together with large-scale pretraining on biomedical datasets, cloud-based computing infrastructures, and open-source frameworks, AutoML has significantly accelerated the translation of AI tools into healthcare [23,24].

Despite these limitations, the study has important strengths. It is, to our knowledge, the first head-to-head comparison of multiple LLM-based chatbots in orthopedics, jointly assessing reliability, usefulness, quality, and readability. The use of a structured methodology, independent expert raters, and multiple validated readability indices enhances the robustness of the findings. By systematically characterizing the trade-off between accuracy and readability, this study provides a foundation for future development and clinical integration of AI-based patient education tools.

## 5. Conclusions

In this comparative evaluation of three AI chatbot models for patient education on RC tears, Gemini 1.5 Flash and ChatGPT-4o provided the most reliable, useful, and high-quality information, whereas DeepSeek-V3 generated the most readable content. No single model achieved an optimal balance between accuracy and readability, underscoring the need for clinician oversight and careful integration into patient education.

These findings suggest that hybrid approaches—such as layered information delivery or combined use of different chatbots—may enhance patient communication by ensuring both factual accuracy and linguistic accessibility. Future work should focus on incorporating health literacy principles into model development, testing comprehension in real-world patient populations, and addressing the ethical and legal frameworks necessary for safe implementation. Ultimately, AI chatbots hold promise as adjunctive tools for orthopedic patient education, provided that their outputs are critically evaluated and adapted by healthcare professionals.

## Figures and Tables

**Table 1:** Patient Questions on Rotator Cuff Tears.

Category	No	Question
Diagnosis and Symptoms	1	What is a rotator cuff tear?
	2	What are the symptoms of a shoulder tendon tear?
	3	How can I tell if I have a tear in my shoulder?/How is it detected?
	4	Is MRI necessary to detect a shoulder tear? Can it clearly show the tear?
	5	What is a partial tear in the shoulder, and how does it differ from a complete tear?
	6	If I have shoulder pain but can still move my arm, could it still be a tear?
	7	How can a shoulder tendon tear be distinguished from a muscle strain?
Treatment and Interventions	8	Can a rotator cuff tear heal without surgery?/Can it be treated non-surgically?
	9	Is physical therapy sufficient for treating a rotator cuff tear?
	10	When is surgery necessary for shoulder tendon tears? Does every tear require an operation?
	11	How is rotator cuff surgery performed?
	12	What is the difference between arthroscopy and open surgery for shoulder tendon tears?
	13	Is there pain after shoulder tendon tear surgery?
	14	Is postoperative physical therapy necessary, and what does the process involve?
	15	What happens if a shoulder tendon tear is left untreated? Can it cause permanent damage?
	16	Are PRP or stem cell therapies effective for shoulder tendon tears?
	17	What are the risks of shoulder tendon tear surgery?
Lifestyle and Activity	18	When can I use my arm after rotator cuff surgery?
	19	When can I return to sports?/Can I play sports again?
	20	Can a shoulder tendon tear reoccur, and how can it be prevented?

**Table 2:** Inter-rater reliability (ICC) of AI-generated responses across models and evaluation criteria (higher ICC = stronger agreement among raters).

Model	Criterion	ICC (95% CI)	Interpretation
ChatGPT-4o	Reliability	0.900 (0.758–0.959)	Excellent agreement
	Usefulness	0.848 (0.691–0.934)	Good–excellent
	GQS	0.726 (0.469–0.878)	Good
Gemini 1.5 Flash	Reliability	0.897 (0.767–0.957)	Excellent agreement
	Usefulness	0.776 (0.559–0.901)	Good
	GQS	0.856 (0.710–0.937)	Excellent agreement
DeepSeek-V3	Reliability	0.755 (0.401–0.902)	Good
	Usefulness	0.822 (0.472–0.935)	Good–excellent
	GQS	0.743 (0.503–0.886)	Good

ICC = intraclass correlation coefficient. Interpretation thresholds: ≥0.90 = excellent; 0.75–0.89 = good; 0.50–0.74 = moderate. ChatGPT-4o and Gemini achieved excellent agreement for reliability; Gemini had the highest GQS agreement; DeepSeek-V3 showed generally good but slightly lower consistency.

**Table 3:** Reliability, usefulness, and quality (GQS) scores of AI-generated responses on rotator cuff tears.

Criterion	Domain	Model	Mean ± SD	Median (IQR)	η^2^	Test Statistic	*p*-Value	Significant Pairwise Differences
Reliability	Diagnosis & Symptoms	ChatGPT-4o	4.39 ± 0.80	4.25 (1.0)	0.582	12.47	0.002	Gemini > ChatGPT (*p* = 0.045); Gemini > Deepseek (*p* = 0.003)
		Gemini	5.42 ± 0.59	5.25 (0.0)				
		Deepseek	3.75 ± 0.50	3.75 (0.5)				
	Treatment & Intervention	ChatGPT-4o	4.82 ± 0.58	4.75 (0.94)	0.629	18.98	0.001	Gemini > Deepseek (*p* = 0.003); ChatGPT > Deepseek (*p* = 0.003)
		Gemini	5.57 ± 0.72	5.75 (0.94)				
		Deepseek	3.82 ± 0.44	3.75 (0.44)				
	Lifestyle/Daily Mgmt	ChatGPT-4o	4.25 ± 0.43	4.0 (0.75)	0.624	5.74	0.057	ns (no sig. difference)
		Gemini	6.58 ± 0.28	6.75 (0.5)				
		Deepseek	4.33 ± 0.14	4.25 (0.25)				
	Overall	ChatGPT-4o	4.58 ± 0.67	4.75 (0.88)	0.613	36.95	0.001	Gemini > ChatGPT (*p* = 0.003); Gemini > Deepseek (*p* = 0.003); ChatGPT > Deepseek (*p* = 0.003)
		Gemini	5.67 ± 0.72	5.50 (1.0)				
		Deepseek	3.87 ± 0.46	3.75 (0.5)				
Usefulness	Diagnosis & Symptoms	ChatGPT-4o	5.39 ± 0.71	5.75 (1.0)	0.583	12.50	0.002	Gemini > Deepseek (*p* = 0.024); ChatGPT > Deepseek (*p* = 0.003)
		Gemini	5.82 ± 0.31	5.75 (0.25)				
		Deepseek	4.14 ± 0.78	4.75 (1.5)				
	Treatment & Intervention	ChatGPT-4o	5.35 ± 0.37	5.25 (0.56)	0.679	20.33	0.001	ChatGPT > Deepseek (*p* = 0.003); Gemini > Deepseek (*p* = 0.003)
		Gemini	5.60 ± 0.45	5.75 (0.63)				
		Deepseek	3.90 ± 0.31	3.75 (0.25)				
	Lifestyle/Daily Mgmt	ChatGPT-4o	5.66 ± 0.38	5.75 (0.75)	0.712	6.27	0.044	ns (no sig. difference)
		Gemini	6.16 ± 0.52	6.0 (1.0)				
		Deepseek	4.25 ± 0.0	4.25 (0.0)				
	Overall	ChatGPT-4o	5.41 ± 0.50	5.25 (0.50)	0.658	39.50	0.001	Gemini > Deepseek (*p* = 0.003); ChatGPT > Deepseek (*p* = 0.003)
		Gemini	5.76 ± 0.44	5.75 (0.25)				
		Deepseek	4.03 ± 0.51	3.87 (0.88)				
GQS	Diagnosis & Symptoms	ChatGPT-4o	3.64 ± 0.37	3.75 (0.75)	0.865	17.58	0.001	Gemini > ChatGPT (*p* = 0.003); Gemini > Deepseek (*p* = 0.003); ChatGPT > Deepseek (*p* = 0.009)
		Gemini	4.53 ± 0.26	4.75 (0.5)				
		Deepseek	2.78 ± 0.36	3.0 (0.25)				
	Treatment & Intervention	ChatGPT-4o	3.55 ± 0.38	3.75 (0.63)	0.314	10.49	0.005	Gemini > Deepseek (*p* = 0.018)
		Gemini	4.15 ± 0.73	4.37 (1.19)				
		Deepseek	3.12 ± 0.29	3.12 (0.31)				
	Lifestyle/Daily Mgmt	ChatGPT-4o	3.66 ± 0.38	3.75 (0.75)	0.668	6.01	0.050	ns (no sig. difference)
		Gemini	4.66 ± 0.38	4.75 (0.75)				
		Deepseek	3.41 ± 0.28	3.25 (0.5)				
	Overall	ChatGPT-4o	3.60 ± 0.36	3.75 (0.69)	0.555	33.62	0.001	Gemini > ChatGPT (*p* = 0.003); Gemini > Deepseek (*p* = 0.003); ChatGPT > Deepseek (*p* = 0.003)
		Gemini	4.36 ± 0.58	4.62 (0.5)				
		Deepseek	3.05 ± 0.37	3.0 (0.44)				

**Table 4:** Comparative Analysis of Readability Metrics Across AI Models. (FRE: higher = easier readability; other indices: higher = more complex. Recommended patient materials ≈ 6th–8th grade level).

Metric	Model	Mean ± SD	Median (IQR)	Interpretation	*p*-Value (Pairwise)
**FRE**	ChatGPT-4o	37.0 ± 7.3	37.0 (10.3)	Fairly difficult	Deepseek > ChatGPT (*p* = 0.003); Deepseek > Gemini (*p* = 0.003); ChatGPT > Gemini (*p* = 0.021)
	Gemini	30.7 ± 7.3	31.0 (6.0)	Difficult	
	Deepseek	45.9 ± 7.7	47.5 (9.0)	Closer to plain language	
**FKGL**	ChatGPT-4o	8.9 ± 1.4	8.9 (2.0)	Above target (9th grade)	All comparisons *p* = 0.001
	Gemini	11.6 ± 1.8	11.5 (2.3)	High school level	
	Deepseek	6.5 ± 1.3	6.4 (2.0)	Within target (6th grade)	
**GFI**	ChatGPT-4o	11.1 ± 1.7	11.2 (3.0)	Difficult	Gemini > ChatGPT (*p* = 0.031); ChatGPT > Deepseek (*p* = 0.015); Gemini > Deepseek (*p* = 0.001)
	Gemini	12.5 ± 1.5	12.7 (1.5)	Difficult	
	Deepseek	9.6 ± 1.7	9.5 (2.7)	Easier	
**CLI**	ChatGPT-4o	12.1 ± 1.0	12.1 (1.1)	High school level	Gemini > ChatGPT (*p* = 0.028); Deepseek < both (*p* = 0.001)
	Gemini	13.1 ± 1.2	13.1 (1.6)	High school/college	
	Deepseek	9.6 ± 1.2	9.3 (1.3)	Closer to target	
**SMOG**	ChatGPT-4o	8.7 ± 1.4	8.2 (2.0)	Slightly above target	All comparisons *p* = 0.003
	Gemini	11.1 ± 1.7	11.2 (1.9)	Difficult	
	Deepseek	7.1 ± 0.9	7.0 (1.3)	Within target	
**ARI**	ChatGPT-4o	12.9 ± 1.5	12.8 (2.1)	Difficult	All comparisons *p* = 0.003
	Gemini	17.5 ± 2.6	17.9 (2.8)	Very difficult	
	Deepseek	9.1 ± 2.9	9.6 (4.8)	Easier	

## Data Availability

The datasets generated and analyzed during the current study are openly available without restrictions. Researchers may freely access and use the data for academic purposes.

## Abbreviations

The following abbreviations are used in this manuscript:

GQS	Global quality score
ICC	intraclass correlation coefficient
95% CI	95% Confidence Interval
X	mean
M	median
IQR	interquartile range
SD	standard deviation
